# Molecular epidemiology of bovine tuberculosis in Northern Ghana identifies several uncharacterized bovine spoligotypes and suggests possible zoonotic transmission

**DOI:** 10.1371/journal.pntd.0010649

**Published:** 2022-08-11

**Authors:** Samuel Ekuban Kobina Acquah, Prince Asare, Emelia Konadu Danso, Phillip Tetteh, Amanda Yaa Tetteh, Daniel Boateng, Stephen Osei-Wusu, Theophilus Afum, Yolanda Isabel Ayamdooh, Eric Agongo Akugre, Omar Abdul Samad, Lawrence Quaye, Kwasi Obiri-Danso, Richard Kock, Adwoa Asante-Poku, Dorothy Yeboah-Manu

**Affiliations:** 1 Noguchi Memorial Institute for Medical Research, College of Health Sciences, University of Ghana, Accra, Ghana; 2 Department of Clinical Microbiology, School of Medicine and Health Sciences, University for Development Studies, Tamale, Ghana; 3 Department of Theoretical and Applied Biology, College of Science, Kwame Nkrumah University of Science and Technology, Kumasi, Ghana; 4 Veterinary Services Directorate, Ministry of Food and Agriculture, Tamale, Ghana; 5 Veterinary Services Directorate, Ministry of Food and Agriculture, Bolgatanga, Ghana; 6 Veterinary Services Directorate, Ministry of Food and Agriculture, Wa, Ghana; 7 Department of Biomedical Laboratory Sciences, School of Allied Health Sciences, University for Development Studies, Tamale, Ghana; 8 Department of Pathobiology and Population Sciences, Royal Veterinary College, London, United Kingdom; Foshan University, CHINA

## Abstract

**Objective:**

We conducted an abattoir-based cross-sectional study in the five administrative regions of Northern Ghana to determine the distribution of bovine tuberculosis (BTB) among slaughtered carcasses and identify the possibility of zoonotic transmission.

**Methods:**

Direct smear microscopy was done on 438 tuberculosis-like lesions from selected cattle organs and cultured on Lowenstein-Jensen media. Acid-fast bacilli (AFB) isolates were confirmed as members of the *Mycobacterium tuberculosis* complex (MTBC) by PCR amplification of IS*6110* and rpo*ß*. Characterization and assignment into MTBC lineage and sub-lineage were done by spoligotyping, with the aid of the SITVIT2, miruvntrplus and *mbovis.org* databases. Spoligotype data was compared to that of clinical *M*. *bovis* isolates from the same regions to identify similarities.

**Results:**

A total of 319/438 (72.8%) lesion homogenates were smear positive out of which, 84.6% (270/319) had microscopic grade of at least 1+ for AFB. Two hundred and sixty-five samples (265/438; 60.5%) were culture positive, of which 212 (80.0%) were MTBC. Approximately 16.7% (34/203) of the isolates with correctly defined spoligotypes were negative for IS*6110* PCR but were confirmed by rpo*ß*. Spoligotyping characterized 203 isolates as *M*. *bovis* (198, 97.5%), *M*. *caprae* (3, 1.5%), *M*. *tuberculosis* (Mtbss) lineage (L) 4 Cameroon sub-lineage, (1, 0.5%), and *M*. *africanum* (Maf) L6 (1, 0.5%). A total of 53 unique spoligotype patterns were identified across the five administrative regions (33 and 28 were identified as orphan respectively by the SITVIT2 and *mbovis.org* databases), with the most dominant spoligotype being SIT1037/ SB0944 (77/203, 37.93%). Analysis of the bovine and human *M*. *bovis* isolates showed 75% (3/4) human *M*. *bovis* isolates sharing the same spoligotype pattern with the bovine isolates.

**Conclusion:**

Our study identified that approximately 29% of *M*. *bovis* strains causing BTB in Northern Ghana are caused by uncharacterized spoligotypes. Our findings suggest possible zoonotic transmission and highlight the need for BTB disease control in Northern Ghana.

## Author summary

Bovine tuberculosis (BTB) is a disease of global public and animal health significance. The disease is endemic in major livestock producing countries in sub-Saharan Africa. Little or no attention is given to the disease and its impact on livestock and humans. We conducted a cross sectional molecular epidemiology study in Northern Ghana to understand the population structure and distribution of tuberculosis in cattle and to assess the possibility of transmission of the disease to humans. We collected tuberculosis-like lesions from selected cattle organs from three regional abattoirs in the five administrative regions of Northern Ghana and found half of the total samples to be infected with *Mycobacterium bovis*. Majority of our isolates appeared to be uncharacterised suggesting the need for routine tracking and characterisation of the *M*. *bovis* species circulating in the sub-region. Comparison of our analysis of the *M*. *bovis* from bovine origin to that obtained from human *M*. *bovis* from the same geographical area showed 75% (3/4) of the human *M*. *bovis* isolates having same molecular pattern with the bovine isolates. Our findings suggest possible zoonotic transmission and highlight the need for BTB disease control in Northern Ghana.

## Introduction

Tuberculosis (TB) is a chronic granulomatous infectious disease of One Health importance. The World Health Organisation (WHO) estimates indicate that 10 million individuals comprising 5.6 million men, 3.2 million women, and 1.2 million children less than 15 years fell ill with TB, while 1.2 million individuals died in 2019 [1]. The causative agents of tuberculosis in mammals are homogenetic acid-fast bacteria referred to as the *Mycobacterium tuberculosis* complex (MTBC). Members within the complex show preference for specific mammalian hosts. *M*. *tuberculosis sensu stricto* (Mtbss) and *M*. *africanum* (Maf) are responsible for human tuberculosis [2,3]. The animal adapted members of the complex comprise *M*. *bovis*, *M*. *caprae*, *M*. *bovis* BCG, *M*. *microti*, *M*. *pinnipedii*, *M*. *origys*, *M*. *mungi*, *M*. *suricattae*, the dassie bacillus, and the chimpanzee bacillus which are widely distributed in domesticated animals and wildlife [4–7]. Notwithstanding, animal adapted species such as *M*. *bovis* and *M*. *orygis* occasionally cause zoonotic tuberculosis [8,9]. Globally, the actual number of zoonotic TB remains unknown, with the WHO [10] estimating 147,000 cases of zoonotic TB and 12,500 deaths occurring in 2016 [11,12].

Zoonotic transmission primarily occurs through close contact with infected cattle, consumption of contaminated cattle products, or unpasteurized milk and milk products [13]. Therefore, the burden of zoonotic TB reflects the prevalence and distribution of the disease in cattle, which serves as an important reservoir and further highlights cattle to human transmission through contaminated cattle products [14].

In resource-limited countries, there are limited information on routine surveillance of field and abattoir investigations of BTB. Protocols are either absent, inadequate or not enforced. The existing strategy is to conduct on-site post-mortem macroscopic examination of carcasses by veterinary staff and sanitary inspectors in abattoirs to remove potentially infected carcasses (gross pathologic lesions) without adequate laboratory support making the strategy unreliable and inefficient [15]. There is, therefore, limited information on the prevalence and distribution of BTB, especially in sub-Saharan Africa, where the disease is enzootic in significant livestock-producing countries [16,17].

Previous studies among pulmonary TB patients in Ghana revealed that zoonotic TB is significantly higher in clinical cases from northern Ghana [9, 18]. The studies further indicated that zoonotic TB is associated with patients who have had contact with livestock or unpasteurized milk and milk products [9,18].

To better understand the population structure and distribution of MTBC from livestock in the northern part of Ghana, an abattoir-based cross-sectional molecular epidemiology study was conducted within three regional abattoirs. We sought to characterize MTBC isolates from animal origin and assess the possibility of zoonotic transmission of *M*. *bovis* by investigating isolates from humans in the same geographical area and within the same period.

## Methods

### Ethics statement

The Scientific and Technical Committee (STC) and the Institutional Review Board (IRB) of the Noguchi Memorial Institute for Medical Research (NMIMR), University of Ghana, with a federal wide assurance number FWA00001824, reviewed and approved the protocols (protocol number 070/19-20) and procedures for this study.

### Study area/site

The study was conducted in five administrative regions of Northern Ghana, comprising Upper East, Upper West, North East, Savanna, and Northern regions. The regions share boundaries with Cote D’Ivoire in the west, Togo in the east, and Burkina Faso in the north. The regions are predominantly low-lying grassland, interspersed with guinea savannah woodland. The inhabitants are mostly livestock and cereal farmers producing more than 70% and 80% of Ghana’s cereal and livestock, respectively [19,20]. Three primary livestock production systems (intensive, semi-intensive, and extensive) are practiced within the region. In all the regional capitals, there are abattoirs manned by trained veterinary personnel [21]. These abattoirs receive large and small ruminants from surrounding livestock markets and neighbouring West African countries (Burkina Faso, Mali, Niger, and Togo), which are slaughtered, and carcasses sold in the markets.

### Sampling

**Fig 1:**
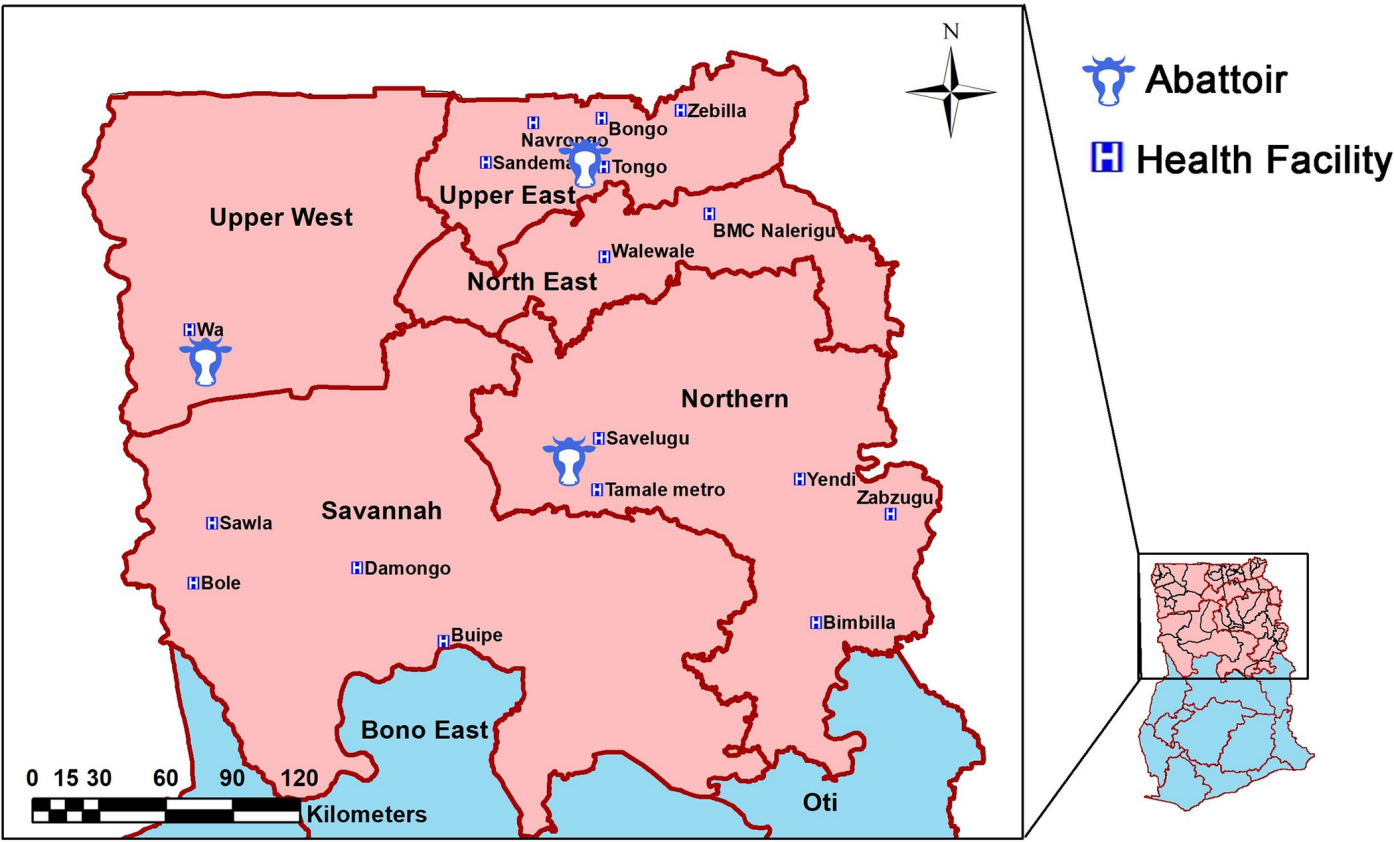
Study area and sampling sites. The base map as well as the copyright information for use of the base map files can be obtained at https://data.humdata.org/dataset/cod-ab-gha. No changes were made to the base map files.

Sampling of carcasses was done at the three regional abattoirs (Bolgatanga, Wa, and Tamale Abattoir) with the help of certified veterinary personnel (Fig 1).

**Fig 2:**
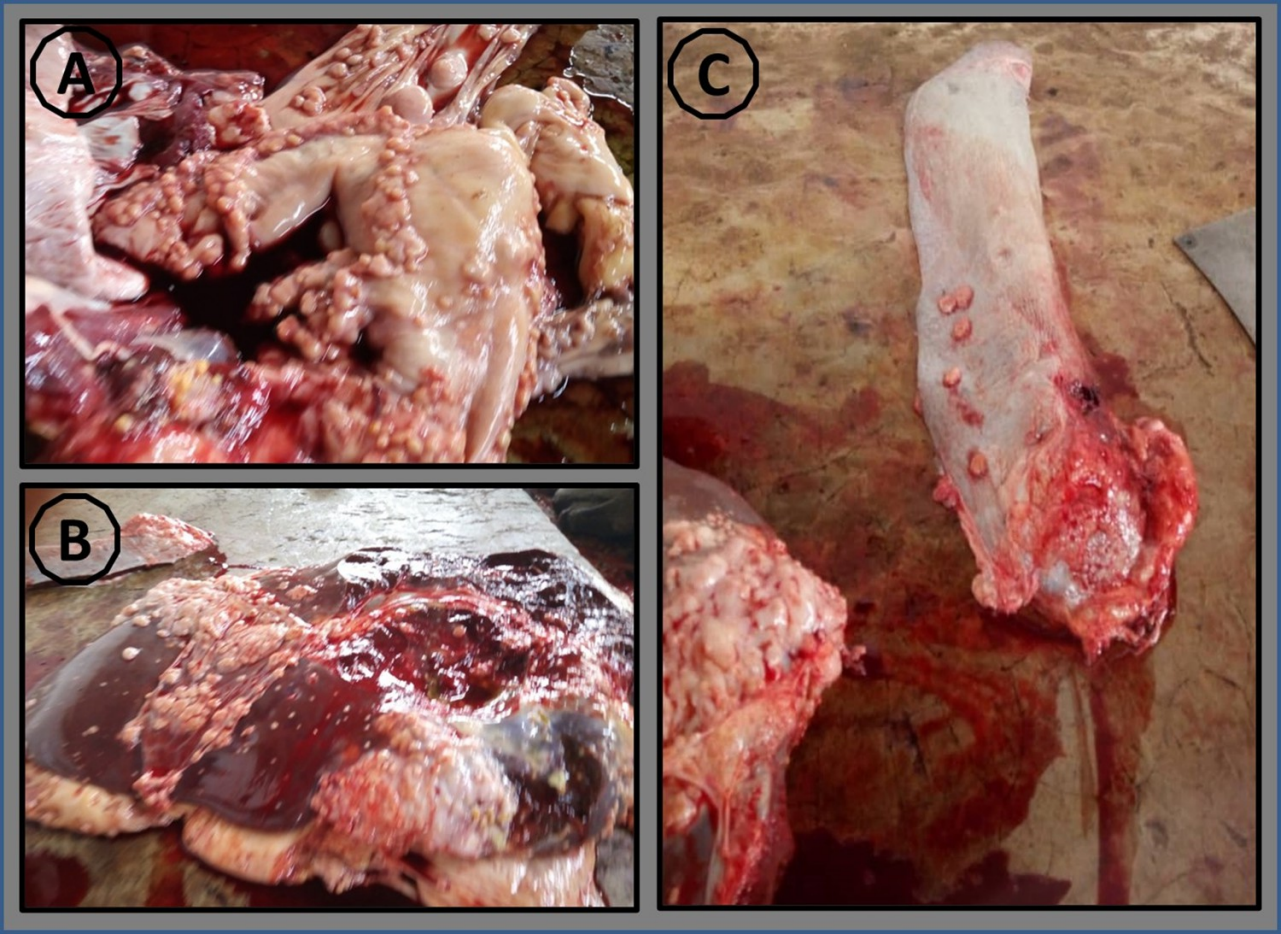
Granulomatous macro lesions sampled from selected cattle organs: (A) granulated lesion from the lung (B) granulated lesion from the liver (C) granulated lesion from the spleen.

Following routine meat inspection by officers of Veterinary Service Department (VSD), granulomatous lesions suggestive of BTB were consecutively sliced into sterile 50 mL Falcon tubes and appropriately labeled with unique IDs (Fig 2). The labeled samples were tightly capped, sealed with parafilm, packaged, and transported on ice according to the WHO guidance on regulations for the transport of infectious substances [22] to the NMIMR laboratory for analysis.

### Laboratory methods

All laboratory processes were performed aseptically in a biosafety level three physical containment laboratory (BSL-3) with a Class II biosafety cabinet (Medical air Technology, Manchester, UK) taking into cognizance all necessary Good Laboratory Practices (GLP) and safety protocols codified by the NMIMR for the processing of such samples. To avoid laboratory cross contamination, human and bovine samples were processed in separate BSL-3 laboratories.

### Nodule homogenization

The lesions were carefully examined in the laboratory. A sterile scalpel was used to remove all adipose and other non-pathological tissues to expose the characteristic thick solid granulomatous nodule. The nodules were incised and discrete tubercles manually homogenized in a sterile mortar with a sterile pestle. The homogenized tubercles were suspended in sterile phosphate buffered saline (PBS) in 50 ml Falcon tube for decontamination. Five millilitres (5.0 ml) of each tissue homogenate suspension were decontaminated with equal volumes of 5% (w/v) oxalic acid in labelled 50 mL Falcon tubes with intermittent vortexing at room temperature for 30 minutes following the protocol described by Yeboah-Manu *et*. *al*. [23]. The suspensions were concentrated by centrifugation at 4000 rpm for 30 minutes to pellet the mycobacteria. The supernatant was carefully decanted and the pellets were re-suspended in 2.0 ml of PBS for smear microscopy and inoculation on to Lowenstein- Jensen (LJ) culture media.

### Microscopy

Smears were prepared directly from the decontaminated homogenate pellet on clean labeled slides, allowed to air dry, heat fixed and stained with the Ziehl-Neelsen method for acid-fastness. The staining processes were controlled with known positive and negative smears [24].

### Mycobacterial isolation

One hundred microliters (100 μl) of the decontaminated tissue homogenate was inoculated into four (4) labelled Lowenstein- Jensen (L-J) media slants; two supplemented with glycerol and the other two supplemented with 0.4% sodium pyruvate [23,25,26]. The inoculated media slants were incubated at 37°C for 12 weeks for the appearance of confluent macroscopic growth of mycobacteria [25]. Colonies suggestive of mycobacteria were quantified using the semi-quantitation grading procedure for bacterial isolates on growth media with a numerical designation as 3+, 2+, 1+, scanty based on the number of colonies identified in streak area [27]. The colonies were stained by the Ziehl-Nielsen method for acid fastness [28,29].

### Species identification and strain differentiation

A loopful of all acid-fast isolates was inactivated at 95°C for 1 hour in sterile distilled water and DNA extracted for molecular analysis as previously described [30]. Members of MTBC were confirmed by PCR amplification of MTBC-specific insertion sequence 6110 (IS*6110*) and rpo*β* [31–33]. All confirmed MTBC isolates were further characterized by spoligotyping using appropriate primers; DRa (5’-CCG AGA GGG GAC GGA AAC-3’) and biotinylated DRb (5’-GGT TTT GGG TCT GAC GAC-3’) and protocols as previously described [2]. Using the spoligotype data and the *Mycobacterium bovis* spoligotype database (*mbovis.org*) [34] the isolates were identified and SB numbers assigned. New spoligotyping patterns identified from this study were submitted to *mbovis.org* for generation of corresponding SB numbers and subsequent inclusion in the database. For the purposes of comparing spoligotyping patterns between animal and human derived isolates as well as assigning lineages, sub-lineages, and their shared international type (SIT) numbers, the spoligo patterns were uploaded into the MIRU-VNTR*plus* web database (http://www.miru-vntrplus.org) [35,36] for identification by similarity search. The SITVIT2 web database (http://www.pasteur-guadeloupe.fr:8081/SITVIT2) [37] was additionally used to complement the MIRU-VNTRplus database. All genotyping assays were controlled by including H37Rv and *M*. *bovis* BCG DNA as positive controls and nuclease free water as a negative control.

### Phylogenetic analysis of MTBC isolates retrieved from both bovine and human source

The online MIRU-VNTR*plus* database was used for clustering analysis and phylogenetic reconstruction. The MTBC spoligotypes from bovine origin were compared to *M*. *bovis* spoligotypes obtained from our previous clinical study conducted in the same region [38] for phylogenetic analysis. We employed the default parameters using the categorical parameter and the unweighted pair group method with arithmetic mean (UPGMA) coefficient to reconstruct our phylogenetic tree.

### Data analysis

Demographic and clinical data of each livestock was collected, entered into Microsoft Access database, and validated to correct for errors and double entries. Descriptive statistics were carried out for both the categorical and numerical variables. Cross-tabulations were employed to explore the relationship between the different outcomes and selected variables using Chi-square and student t-test where applicable. Where appropriate, the Fisher’s exact or the chi-square tests and logistic analysis were used to assess statistical significance. A p-value less than 0.05 at 95% confidence level was considered significant. All statistical analyses were performed in Stata version 14. The ArcMap tool employed in ArcGIS (Economic and Social Research Institute, version 10.1) [39] was used for map construction. The base maps used are freely available at https://data.humdata.org/dataset/cod-ab-gha.

## Results

### Observed activities at a typical abattoir

**Fig 3:**
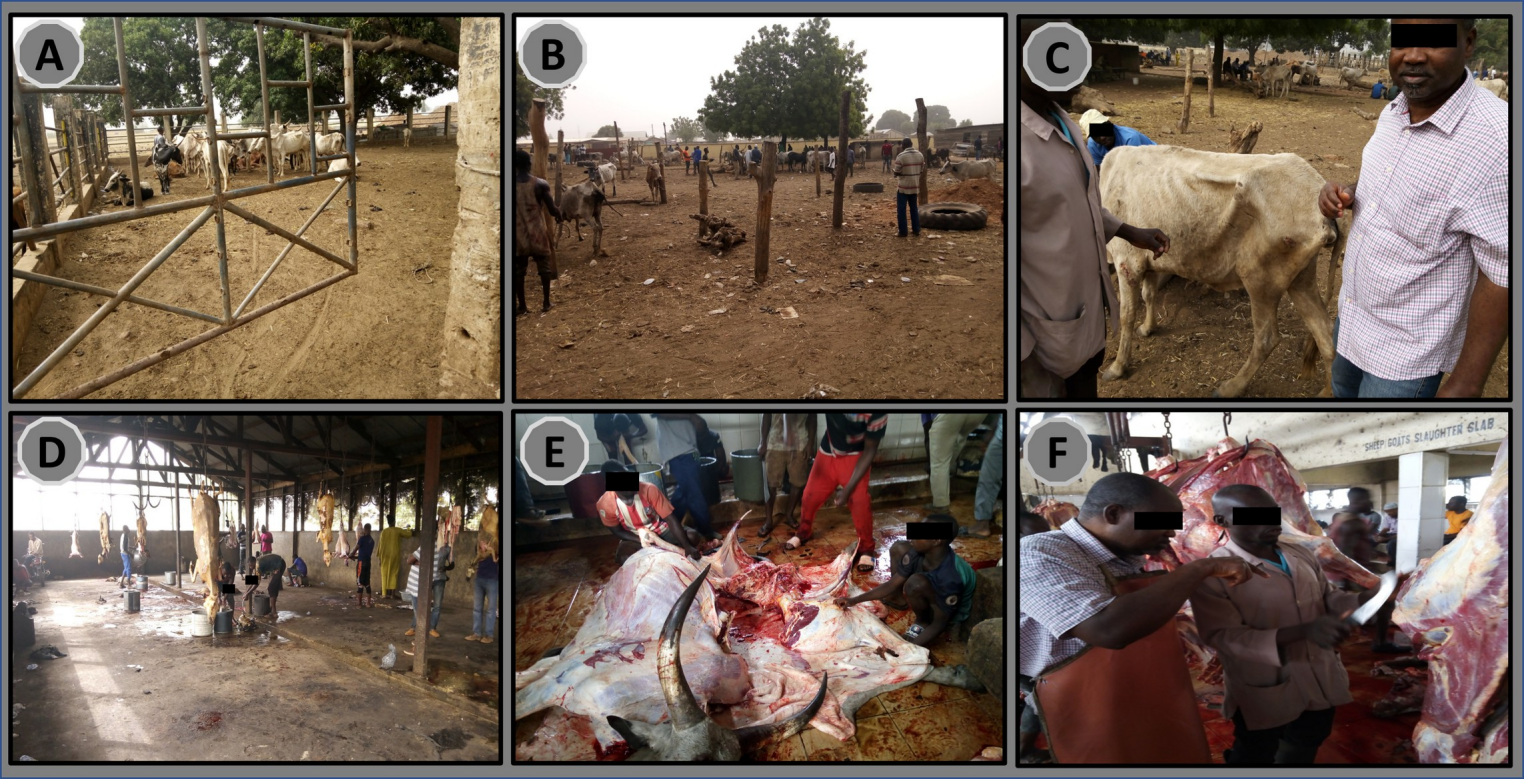
Activities at a typical abattoir. (A and B) lairage for cattle (C) ante-mortem inspection by a veterinary officer (D) slaughterhouse for small ruminant (E) slaughterhouse for large ruminant (F) Post-mortem inspection for macro lesions by a veterinary officer.

We found activities performed at a typical abattoir in the region to include lairage for the livestock (Fig 3A and 3B), where temporary ante-mortem inspections (Fig 3C) are carried out and then proceeded to slaughter. Small (Fig 3D) and large ruminants (Fig 3E) are slaughtered at the abattoir, following which a veterinary officer performs a post-mortem inspection for macro lesions (Fig 3F). Judgment is made whether the meat is fit for consumption or otherwise marked as condemned. Data from one abattoir showed that an average of 27 cattle were slaughtered each day, of which 2 (7.4%) had a tuberculosis-like lesion on at least one internal organ.

### Physical and demographic characteristics of livestock sampled

**Fig 4:**
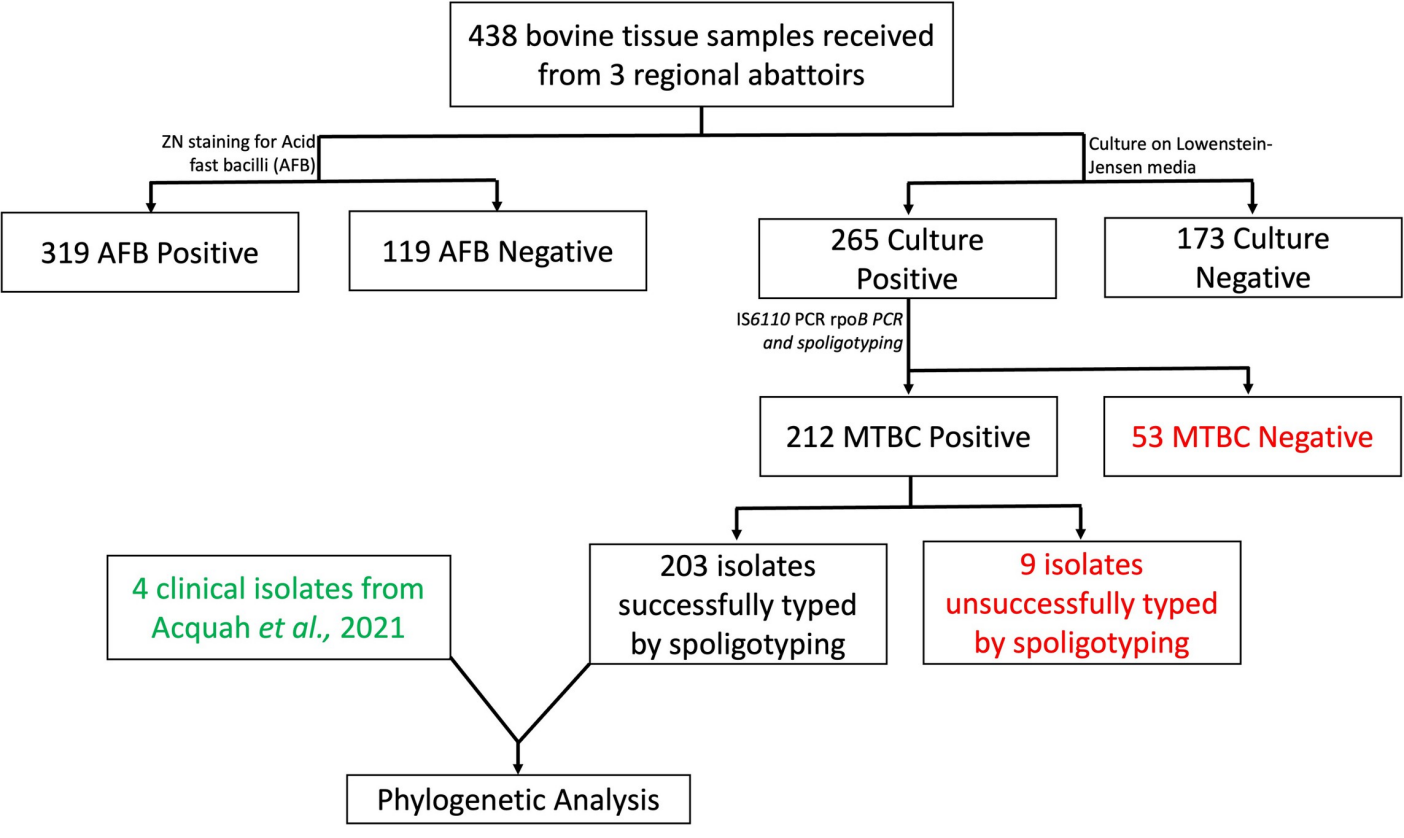
Analysis workflow. MTBC: *Mycobacterium tuberculosis* complex.

Four hundred and sixty-eight (468) bovine tissue samples were collected from the three abattoirs for laboratory analysis (Fig 4), of which 30 were excluded from further analysis due to lack of appropriate demographic data accompanying the samples. The animals were brought from all over Northern Ghana although they were slaughtered at the three regional abattoirs: Bolgatanga abattoir (N = 8), Tamale abattoir (N = 401), and Wa abattoir (N = 29). Of the 438 samples, post-mortem examination data on the type of pathology was unavailable for 141 samples. The remaining 297 had records of a post-mortem examination, indicating 16.5% (49/297) abscesses, 37.7% (112/297) nodules, and 45.8% (136/297) both nodules and abscesses. Both pulmonary and extra-pulmonary samples were collected. Most of the samples were from lungs only (59.6%, 261/438), with the remaining coming from extra-pulmonary sites such as lymph node, liver, spleen, kidney, heart, or multiple sites (Table 1).

**Table 1:** Infected sites/organs and region where samples were obtained.

Site of infection	North East (N = 15)	Northern (N = 320)	Savannah (N = 66)	Upper East (N = 8)	Upper West (N = 29)	Total, n (%) (N = 438)
Heart	0	1	0	0	0	1 (0.2)
Kidney	0	0	1	0	0	1 (0.2)
Liver	0	10	5	0	4	19 (4.3)
Lung	11	197	37	3	13	261 (59.6)
Lymph node	2	15	5	0	1	23 (5.2)
Spleen	0	2	0	1	0	3 (0.7)
Multiple organs, including lungs	2	80	17	3	9	111 (25.3)
Multiple organs, excluding lungs	0	15	1	1	2	19 (4.3)

**Fig 5:**
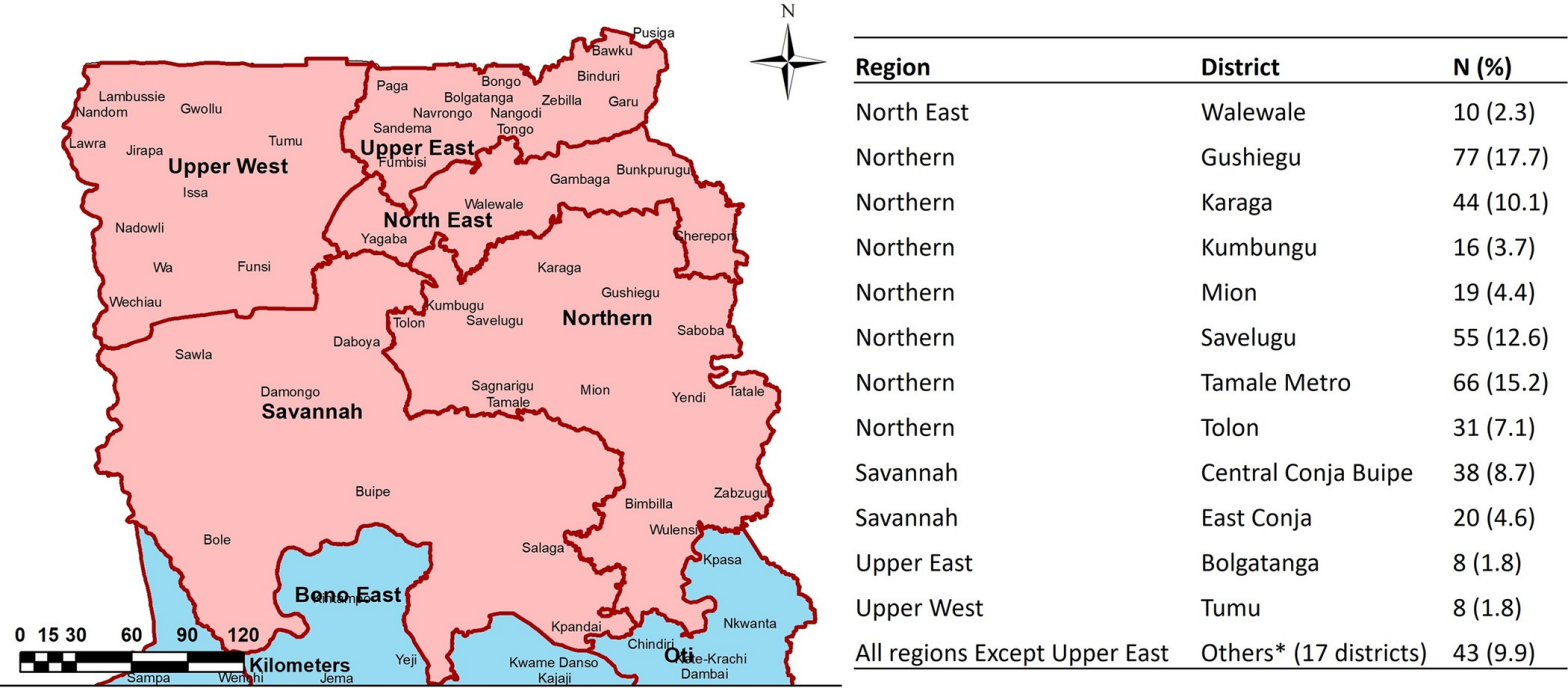
Geographical origin of slaughtered cattle. Animals sampled came from 29 districts within Northern Ghana. The base map as well as the copyright information for use of the base map files can be obtained at https://data.humdata.org/dataset/cod-ab-gha. No changes were made to the base map files.*Other districts include: Dabo, Damongo, East_Mamprusi, Gulu, Hamile, Janga, Kabulpe, Kpalbei, Laura, Nalerigu, Nanton, Sagnerigu, Sissala West, Sissala North, West Gonja, Wa West, and West Mamprusi.

Majority of cattle sampled were of the West African Short Horn (WASH) breed (80.0%, 307/384 [data missing for 54 animals]) and suffered from pulmonary infections (S1 Fig). Other breeds of cattle included Sanga (52), Ndama (16), Fulani white (4), Zebu (3), and Gudaly (2) (S1 Fig). The median age of the cattle was seven years (IQR, 5–8 years) (S2 Fig). The majority were females (70.7%, 307/434 [data missing for 4 animals]). There was no observed statistical difference between the mean age of male cattle (6.5yrs) versus female cattle (6.6yrs) (p = 0.732). Cattle slaughtered in the three abattoirs came from 29 districts of the five administrative regions of Northern Ghana (Fig 5).

### Pyruvate supports *M*. *bovis* growth better than glycerol supplemented media

**Fig 6:**
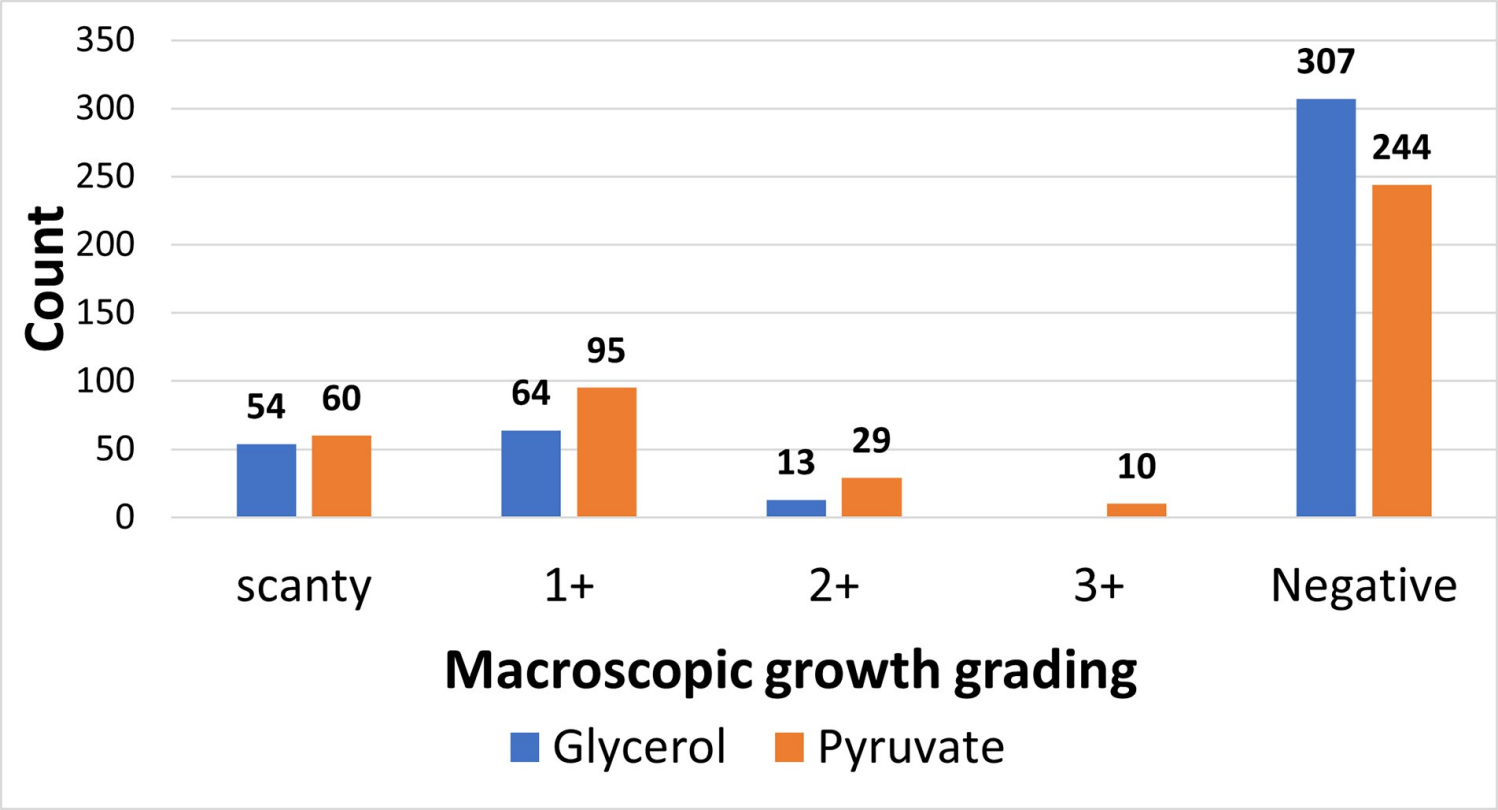
Degree of macroscopic growth on LJ media. The average macroscopic grading per each pair of LJ tube was used as count data for each cultivated sample.

All 438 homogenized samples were examined directly for acid-fast bacilli (AFB). In all, 72.8% (319/438) were AFB positive, with greater than 60% being smear grade of at least 1+ (Table 2). Two hundred and sixty-five samples (265/438; 60.5%) yielded mycobacteria growth. Compared to the Tamale abattoir, samples from the Bolgatanga and Wa abattoirs were 2 times more likely to be culture positive (p<0.05). Also, with respect to origin of sample, compared to the Northern region, samples from Upper West were 3 times more likely to be culture positive (OR = 2.9, p = 0.024). All other logistic regression analysis involving site of infection, organ source and district of origin were not statistically significant. Of the 319 AFB positive samples, 16.9% (54/319) were culture negative. There were significantly more isolates on pyruvate-containing media compared to glycerol-containing media (p<0.001) (Fig 6).

**Table 2:** Microscopic and culture examination of sample homogenates.

Examination	Frequency n (%) N = 438
**Direct Microscopy**	
scanty	49 (11.2)
1+	82 (18.7)
2+	65 (14.8)
3+	123 (28.1)
Negative	119 (27.2)
**Culture**	
Positive	265 (60.5)
Negative	173 (39.5)

### Population structure and spatial distribution of bovine MTBC in Northern Ghana

**Fig 7:**
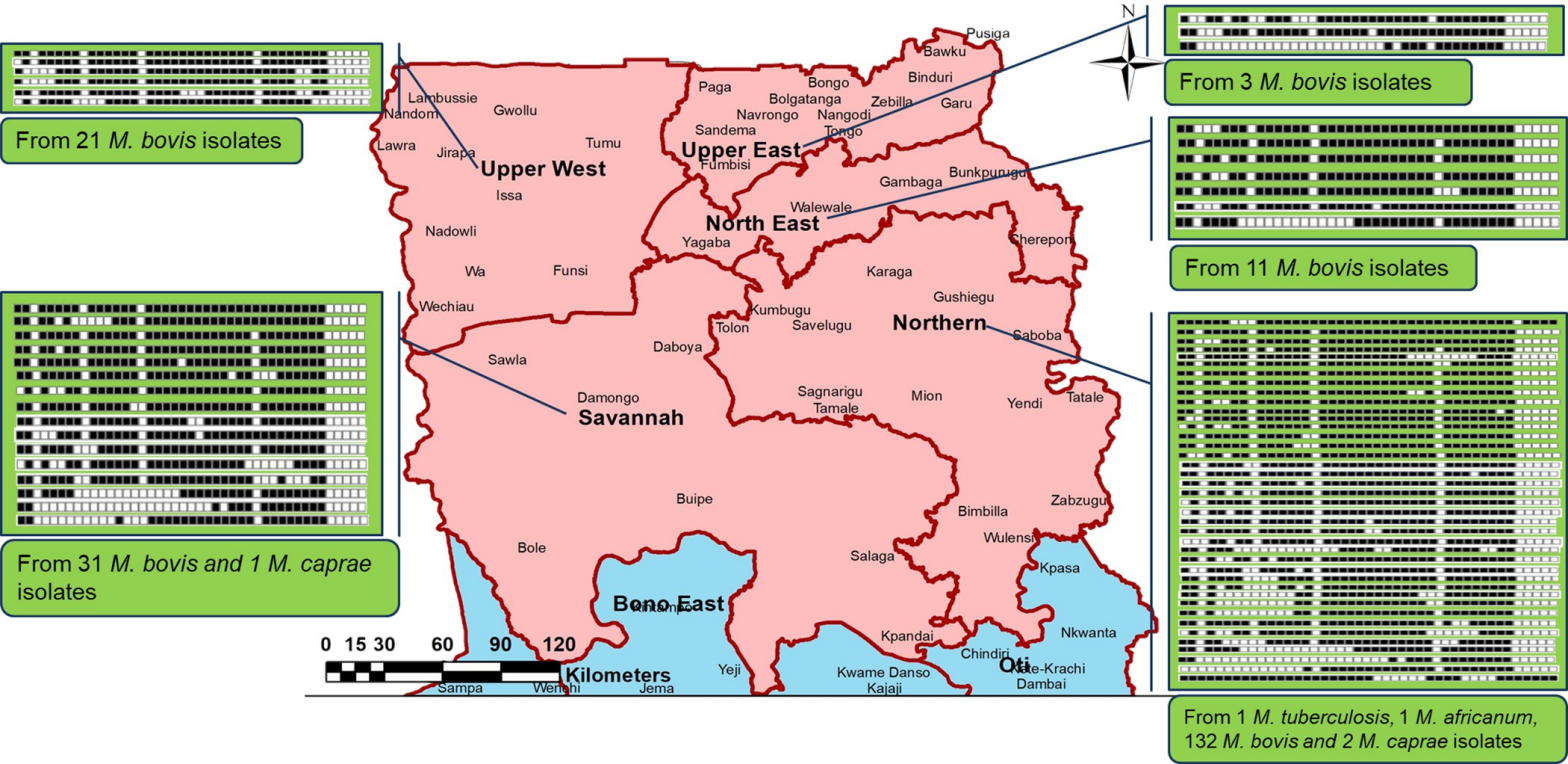
Spatial distribution of spoligotypes. The base map as well as the copyright information for use of the base map files can be obtained at https://data.humdata.org/dataset/cod-ab-gha. No changes were made to the base map files.

Out of the 265 mycobacteria culture positives, 212 were confirmed as members of MTBC. Analysis of the spoligotypes by SITVIT2 and MIRU-VNTR*plus* databases, identified; *M*. *bovis* (97.5%, 198/203), *M*. *caprae* (1.5%, 3/203), *M*. *tuberculosis* (Lineage (L) 4 Cameroon sub-lineage, 0.5%, 1/203), and *M*. *africanum* (L6, 0.5%, 1/203). Nine isolates were not successfully typed by spoligotyping. Approximately 16.7% (34/203) of the isolates were found to be negative for IS*6110* PCR but confirmed as MTBC by rpo*B* and spoligotyping. The study identified 53 unique (distinct) spoligotype patterns (out of which 33 were orphan by MIRU-VNTR*plus* database and 28 uncharacterized by *mbovis.org* database), with the most dominant spoligotype being SIT1037/SB0944 (77/203, 37.93%) (Table 3). Cattle from which *M*. *caprae* were isolated came from the Northern and Savannah regions (Fig 7 and S1 Table). The Northern region had the most diverse spoligotypes (Fig 7 and S1 Table). Our findings show that BTB in Northern Ghana is mainly characterized by infection with *M*. *bovis*, with less than 1% of the BTB burden caused by the human-adapted species (Maf and Mtbss).

**Table 3:** Identified spoligotype patterns.

	SIT*	SB^#^	Species	Frequency n (%)	Spoligotype Binary Pattern
1	181	SB0147	*M*. *africanum*	1 (0.49)	1111110001111111111111111111111111111101111
2	482	SB0120	*M*. *bovis*	2 (0.98)	1101111101111110111111111111111111111100000
3	665	SB0134	*M*. *bovis*	4 (1.97)	1100011101111110111111111111111111111100000
4	683	SB0140	*M*. *bovis*	1 (0.49)	1101101000001110111111111111111111111100000
5	757	SB0328	*M*. *bovis*	1 (0.49)	1101111101011110111111111111101111111100000
6	1032	SB0878	*M*. *bovis*	1 (0.49)	1101111101111110111111111100000000111100000
7	1036	SB0850	*M*. *bovis*	3 (1.47)	1101111101111110111111111111100111111100000
8	1037	SB0944	*M*. *bovis*	77 (37.93)	1101111101111110111111111111101111111100000
9	1603	SB0300	*M*. *bovis*	26 (12.81)	1101101101111110111111111111101111111100000
10	2813	SB1027	*M*. *bovis*	5 (2.46)	1101111101111110111111111100101111111100000
11	2840	SB1026	*M*. *bovis*	4 (1.97)	1101001101111110111111111111101111111100000
12	2842	SB0951	*M*. *bovis*	1 (0.49)	1101111101111110111111111111101111110100000
13	3025	SB1025	*M*. *bovis*	1 (0.49)	1101111101111110111111111111100011111100000
14	3539	SB1275	*M*. *bovis*	1 (0.49)	1101111101111110111101111111101111111100000
15	3720	SB1099	*M*. *bovis*	1 (0.49)	1001111101111110111111111111101111111100000
16	3735	SB1431	*M*. *bovis*	1 (0.49)	1101111101111110111111111101100011111100000
17	3736	SB1432	*M*. *bovis*	3 (1.47)	0101001101111110111111111111101111111100000
18	3742	SB1439	*M*. *bovis*	4 (1.97)	1101111101111100111111111111101111111100000
19	3743	SB1440	*M*. *bovis*	1 (0.49)	1101111101111000111111111111101111111100000
20	3760	SB1410	*M*. *bovis*	2 (0.98)	1001101101111110111111111111101111111100000
21	Orphan or New-1	SB2738^+^	*M*. *bovis*	2 (0.98)	1101111001111110111111111111101111111100000
22	Orphan or New-2	SB1418	*M*. *bovis*	1 (0.49)	1101111101111110111111111111101110111100000
23	Orphan or New-3	SB2286	*M*. *bovis*	1 (0.49)	1101101101111010111111111111101111111100000
24	Orphan or New-4	SB2761^+^	*M*. *bovis*	1 (0.49)	1101101001111110111111111111101111111100000
25	Orphan or New-5	SB2762^+^	*M*. *bovis*	5 (2.46)	0101111101111110111111111111101111111100000
26	Orphan or New-6	SB2763^+^	*M*. *bovis*	1 (0.49)	0101101101111110111111111111101111111100000
27	Orphan or New-7	SB1472	*M*. *bovis*	4 (1.97)	1101111101111110111110111111101111111100000
28	Orphan or New-8	SB2764^+^	*M*. *bovis*	2 (0.98)	1101111101111110111110011111101111111100000
29	Orphan or New-9	SB2765^+^	*M*. *bovis*	1 (0.49)	0001101101111110111111111111101111111100000
30	Orphan or New-10	SB2739^+^	*M*. *bovis*	1 (0.49)	1101110000000000001111001111101111001100000
31	Orphan or New-11	SB2740^+^	*M*. *bovis*	1 (0.49)	1101001101011110111111111111101111111100000
32	Orphan or New-12	SB2741^+^	*M*. *bovis*	1 (0.49)	1001111001111010111111111111101111111100000
33	Orphan or New-13	SB2742^+^	*M*. *bovis*	1 (0.49)	1001101100111000111111111111101111111100000
34	Orphan or New-14	SB1517	*M*. *bovis*	4 (1.97)	1100011101111110111111011111111111111100000
35	Orphan or New-15	SB2285	*M*. *bovis*	4 (1.97)	1000011101111110111111111111111111001100000
36	Orphan or New-16	SB2743^+^	*M*. *bovis*	1 (0.49)	1000011101111110111111111111101111001100000
37	Orphan or New-17	SB2744^+^	*M*. *bovis*	3 (1.47)	1101111101111110111100011111101111001100000
38	Orphan or New-18	SB2745^+^	*M*. *bovis*	2 (0.98)	1101111000011110111111111111101111111100000
39	Orphan or New-19	SB2746^+^	*M*. *bovis*	1 (0.49)	1101111000111110111111111111101111111100000
40	Orphan or New-20	SB2747^+^	*M*. *bovis*	1 (0.49)	1101011000011010111111111111101111111100000
41	Orphan or New-21	SB2748^+^	*M*. *bovis*	1 (0.49)	0101111000011110111111111111101111110000000
42	Orphan or New-22	SB2749^+^	*M*. *caprae*	1 (0.49)	1101000000000110111111111110001111111100000
43	Orphan or New-23	SB2750^+^	*M*. *bovis*	1 (0.49)	1101000000000110111111111111001111111100000
44	Orphan or New-24	SB2751^+^	*M*. *bovis*	1 (0.49)	1101000000000110111111111111101111111100000
45	Orphan or New-25	SB2752^+^	*M*. *bovis*	1 (0.49)	1101111101111110111111111111101001111100000
46	Orphan or New-26	SB2753^+^	*M*. *bovis*	2 (0.98)	0101001101111110111111111111000000111100000
47	Orphan or New-27	SB2754^+^	*M*. *bovis*	2 (0.98)	1101100000111110111111111111100011111100000
48	Orphan or New-28	SB2755^+^	*M*. *bovis*	1 (0.49)	1101111001111110111111111111100010001100000
49	Orphan or New-29	SB2756^+^	*M*. *bovis*	6 (2.95)	1101111000000000000011111111101111111100000
50	Orphan or New-30	SB2757^+^	*M*. *bovis*	6 (2.95)	1100000000000000000000001011101111111100000
51	Orphan or New-31	SB2758^+^	*M*. *caprae*	1 (0.49)	0000000000000010111111111111101111111100000
52	Orphan or New-32	SB2759^+^	*M*. *caprae*	1 (0.49)	1100000000001000111111111111101111111100000
53	Orphan or New-33	SB2760^+^	*M*. *tuberculosis*	1 (0.49)	1111111111111111111111000000111100001111111

* SIT: Shared International Type retrieved from SITVIT2 database. All isolates with no available SIT in the SITVIT database were tagged as Orphan or New

# SB: Spoligotype number retrieved from *mbovis.org* database.

^+^ Spoligotype patterns that were newly submitted from this study to *mbovis.org* database.

### Clustering by spoligotyping of *M*. *bovis* from both bovine and human origin

**Fig 8:**
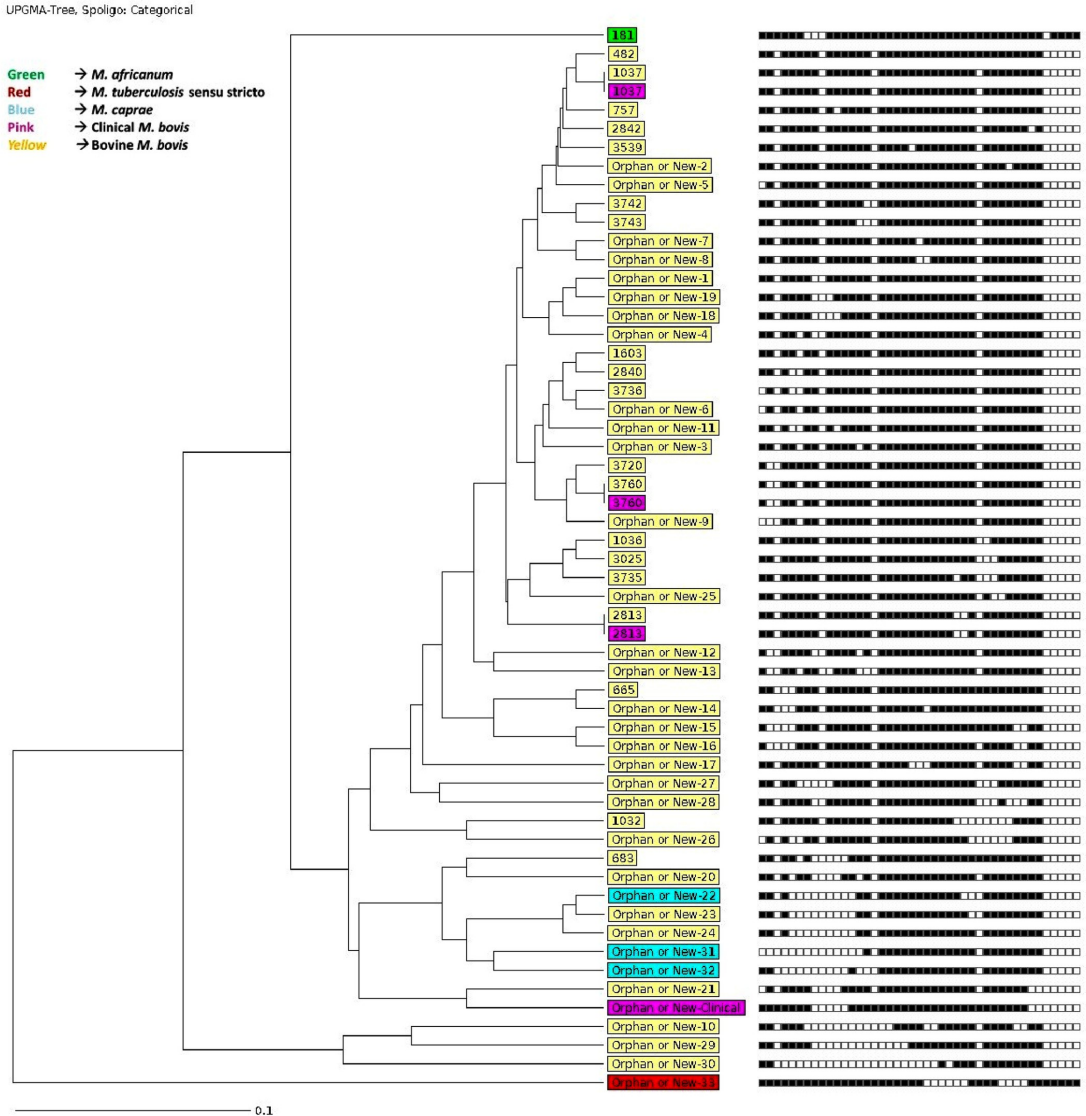
Phylogenetic relationship between *M*. *bovis* isolates from both bovine and humans (clinical). In all, 3 out of the four clinical *M*. *bovis* isolates *(pink)* had the same spoligotype patterns as the bovine isolates. The three *M*. *caprae* with distinct patterns (blue) were isolated from bovine source. Two human-adapted MTBCs were isolated from the bovine source; one Maf L6 (Green) and one Mtbss L4 (Red, Cameroon). The labels on the leaves represent the SIT numbers.

Analysis of spoligotypes obtained from both human and animal origin in the region showed clustering of some spoligotypes (SIT1037/SB0944, SIT3760/SB1410, and SIT2813/SB1027) (Fig 8) [38]. Three of the four human *M*. *bovis* isolates had the same spoligotype patterns as the bovine isolates.

## Discussion

Our objective was to conduct an abattoir-based cross-sectional study in the five administrative regions of Northern Ghana to determine the distribution of bovine tuberculosis (BTB) among cattle carcasses and identify the possibility of zoonotic transmission. Among other significant findings, we identified that, 1) forty-eight percent (212/438) of the lesions were positive for MTBC, 2) the dominant MTBC specie infecting cattle in Northern Ghana was *M*. *bovis* (97.5%, 198/203), 3) there were several uncharacterized spoligotypes, with the most dominant MTBC spoligotype being SIT1037/SB0944 (77/203, 37.9%), 4) there is the possibility of zoonotic BTB transmission in Northern Ghana.

One major challenge facing the livestock sector is condemning edible organs and carcasses to infection [40]. Routine meat inspection at the abattoir by veterinary staff is the sole process that ensures the meat is free from disease and poses no health risk to humans. Data from one abattoir within our study sites showed that an average of 27 cattle were slaughtered each day, of which 2 (7.4%) had a tuberculosis-like lesion on at least one internal organ. This observation is similar to findings from previous studies conducted in other regions of Ghana using tuberculin skin test and serological assays which reported BTB rates ranging between 2.5% and 19% [41–45]. Our observation of a high proportion (48.4%) of the lesions from condemned carcasses (meat not fit for consumption) being positive for MTBC was not surprising as similar observations has been made at the Kumasi abattoir found within the middle belt of Ghana where Shaibu, Atawalna [40] reported that 36.9% of all condemned carcasses were due to *M*. *bovis* infection. This correlates well with the observation that over 80% of the lesions were from the lungs, a typical organ of residence for MTBC. Similar abattoir based observations have been made in other African countries, including Burkina Faso [46,47], Ethiopia [15,48,49] and Côte d’Ivoire [50].

Our study observed that BTB in Northern Ghana is mainly caused by the animal adapted specie, *M*. *bovis* (97.5%, 198/203), with only 1% caused by the human-adapted species (Maf and Mtbss). Interestingly, we also recovered *M*. *caprae* (1.5%), known to infect goats, from the cattle lesions. The isolation of *M*. *caprae* among cattle population could be explained by the sharing of watershed and grazing fields between goats and cattle. This close interaction among these domestic ruminants increases the likelihood of cross-species transmission of mycobacteria [51]. The relatively high *M*. *bovis* recovered from this study could be attributed to the use of 0.5% sodium pyruvate supplemented LJ media in our culture method for isolation, thus, we observed that there were significantly more isolates on pyruvate-containing media compared to glycerol-containing media (p<0.001). It is worth noting that approximately 16.7% (34/203) of the isolates were negative for IS*6110* PCR which is not uncommon for *M*. *bovis* species. This could be due to the lack of the IS*6110* element or inherent mutations at the primer binding site that may have caused false negative results.

The study observed that Northern region recorded the highest cases of BTB with the most diverse spoligotype profile relative to the other regions. Tissue specimens collected were disproportionately higher in the Northern region than in other areas, probably accounting for the high BTB in the region as well as the high spoligotype diversity. Though this may limit comparative analysis between the regions it is worth noting that this observation may be attributable to the increased cattle density and demand for meat in the region. Thus, daily slaughter was relatively higher in the Northern region abattoir. There are several livestock markets including the biggest livestock market in Ghana in Northern region. The markets are opened to livestock farmers from the region and other regions. The main abattoir receives livestock from the livestock markets and kraals within the regions and other West African countries including Burkina Faso, Mali, Niger and Togo. There are possibilities of diverse MTBC strains from cross movement of livestock from one area to another. Increased awareness and continuous epidemiological surveillance of circulating strains of *M*. *bovis* genotypes is recommended for TB control program and tracing of transmission in the region.

*Mycobacterium bovis* is classified into four clonal complexes based on specific deletions (806 to 14,094 bp), SNPs and spoligotypes. The complexes are associated with defined geographical distributions. Clonal complex African 1(Af1) and 2 (Af2) are restricted to Africa, European 2 (Eu2), generally found in the Iberian Peninsula, and European 1(Eu1) are globally distributed [52–55]. Understanding the distribution of these clonal complexes in communities allow for the development of timely interventions, including vaccines, drugs and novel diagnostic tools for the management of BTB and zoonotic TB [56,57]. Spoligotyping analysis of our isolates showed SIT1037/SB0944 as the most dominant (77/203, 37.9%) spoligotype in the region. Other studies have reported similar findings in Mali (40.0%), Cameroon (62.7%), Nigeria (46.0%), and Chad (40.0%) suggesting that the SB0944 variant (a member of the Af1 clonal complex) is prevalent in West Africa [16,58,59]. Generally, BTB in sub-Saharan west-central Africa is mainly caused by the clonal complex Af1, defined by a 5.3-kb deletion of chromosomal DNA (spacer 30) and spoligotype SB0944. It has been suggested that the transhumance cattle movement across the sub region might be attributed to the distribution of SB0944 *M*. *bovis* clonal variants in the region [59,60].

*M*. *bovis* with SIT1603/SB0300 was the second most dominant (26/203 (12.8%)) strain isolated in the region. The SIT1603/SB0300 are subclones of Af1 clonal complex lacking spacer 6. They are however rare or absent in Chad, Nigeria, or Cameroon but widely spread in Mali, where they have been associated with bovine and zoonotic TB [61,62].

The study identified two *M*. *bovis* with SIT 482/SB0120. These strains are members of the BCG family which have not undergone chromosomal deletion but lack spacers 3, 9, 16. They are widely isolated globally, both in humans and livestock [63,64]. In Zambia, 482/SB0120 strains were isolated in cattle and TB patients [65].

Our Spoligotyping analysis further showed 62.3% (33/53) of the isolates had uncharacterized spoligotypes by the MIRU-VNTR*plus* database and 52.8% (28/53) by the *mbovis.org* database. The majority of these uncharacterized strains isolated in Ghana for the first time share characteristics similar to the Af1 clonal complexes. They might have evolved by loss of particular spacers from the progenitor strain, followed a different evolutionary history and spread through cattle populations from different origins and forming the diverse sub clones in the region [16]. This observation suggests a distinct population structure of *M*. *bovis* in the region that requires further studies to characterize and explore the observed diversity. Despite the high rate of BTB with diverse spoligotype profile, there were no clonal complex Africa 2 strains detected in the region. This finding is consistent with reports that suggest that Af2 clonal complexes defined by the deletion RDAf2 and marked by the loss of spoligotype spacers 3 to 7 are rare in West Africa but present at high frequency in East African countries such as Uganda, Burundi, Tanzania, and Ethiopia [60]. The geographical localization of the *M*. *bovis* clonal complexes in Africa may be attributed to the uneven distribution of cattle, cattle density, trading, and movement of cattle [60].

We successfully submitted the 28 unassigned spoligotypes to the *mbovis.org* database and received corresponding SB numbers. Our contribution of spoligotypes to the *m.bovis.org* database is the first from Ghana. These uncharacterized strains may have potentially damaging consequences in case of spillover to humans as the human immune system may not have been exposed to them hence will require more time to fight the new strains.

Phylogenetic analysis of MTBC isolates from this study and previous studies by Acquah *et al*, (2021) in patients with pulmonary tuberculosis showed three out of the four clinical *M*. *bovis* isolates [38] had the same spoligotype pattern as the animal isolates and one (1) *M*. *africanum* (L6) animal isolates had the same spoligotype pattern as the human isolates. The clustering of both clinical and animal spoligotype suggests possible zoonotic and reversed zoonotic transmission. Socioeconomic factors, farming systems, and professional practices could contribute to the transmission of MTBC between humans and livestock in the region. For example, livestock breeding in the region predominantly relies on semi-intensive farming by smallholder farmers within urban and peri-urban communities. The farms are dairy cows and are the primary raw milk and meat supply to cities and towns. Consumption of raw milk and meat products from cattle sometimes slaughtered from unapproved slaughter slabs is common in the region. In the wet seasons, when there is abundant vegetation, cattle, goats and sheep, graze in and around the communities. The close relationship between the communities and cattle offers potential zoonotic transmission between cattle and humans and humans to cattle in areas where human *M*. *tuberculosis* prevalence is very high. Other people at risk of zoonotic transmission are abattoir staff who work with minimal or no understanding of the mode of transmission of zoonotic TB [64,66–68]. Analysis of the spoligotypes identified one (1) *M*. *tuberculosis* (Lineage (L) 4 Cameroon sub-lineage and one (1) *M*. *africanum* (L6) in cattle samples with the *M*. *africanum* (L6) sharing spoligotype profile with human isolates SIT181/ SB0147. Previous studies showed that the Cameroon sub-lineage and the L6 of Maf are the dominant human strains in the region [38]; an indication that livestock in the area is as susceptible to the human-adapted strains as to the animal strains, especially when they are in close proximity and share air space. Similar reverse zoonotic transmissions of *M*. *tuberculosis* have been reported in Nigeria [58], South Africa [69] and Ethiopia [70,71].

We acknowledge a couple of limitations of the study. Firstly, we acknowledge that it is early to predict transmission as the typing tool used (spoligotyping) is not ideal for inferring transmission. However, the proximity coupled with the limited numbers of two out of the three clustered spoligotypes (SIT3760/SB1410 and SIT2813/SB1027) may suggest the same origin. The use of spoligotyping in the study has provided essential insight into the population structure and distribution of *M*. *bovis* in cattle carcasses and the phylogenetic relatedness of *M*. *bovis* isolated in cattle and humans. However, a molecular tool with higher discriminating power will assist the epidemiological analysis of the transmission of *M*. *bovis* strains in cattle and humans. Whole genome sequencing and analysis is the optimum tool needed to confirm zoonotic transmission and we recommend its use in future studies. The relatively low clinical *M*. *bovis* isolates could be attributed to the sputum specimen analyzed for this study. *M*. *bovis* is associated with extra pulmonary TB than pulmonary and therefore extra pulmonary specimen such as lymph node biopsy would have allowed for a better representation of the zoonotic situation in the region.

## Conclusion

Our study identified that approximately 29% of *M*. *bovis* strains causing BTB in Northern Ghana are caused by uncharacterized spoligotypes. Our data suggest possible zoonotic transmission in the region. Data generated will improve BTB disease control and aid in increased awareness in Northern Ghana and the sub-region.

## Supporting information

S1 Fig Breeds and gender distribution of cattle sampled. WASH: West African Short Horn.
(DOCX)

S2 Fig Age distribution of cattle sampled. (DOCX)

S1 Table Supporting demographic and molecular data for 203 spoligotyped isolates. (XLSX)
