# Supplementary figures and images for: Molecular epidemiology of bovine tuberculosis in Northern Ghana identifies several uncharacterized bovine spoligotypes and suggests possible zoonotic transmission
Source: PLoS Negl Trop Dis. 2022 Aug 11;16(8):e0010649. doi: 10.1371/journal.pntd.0010649 (PMC9398027; doi:10.1371/journal.pntd.0010649)

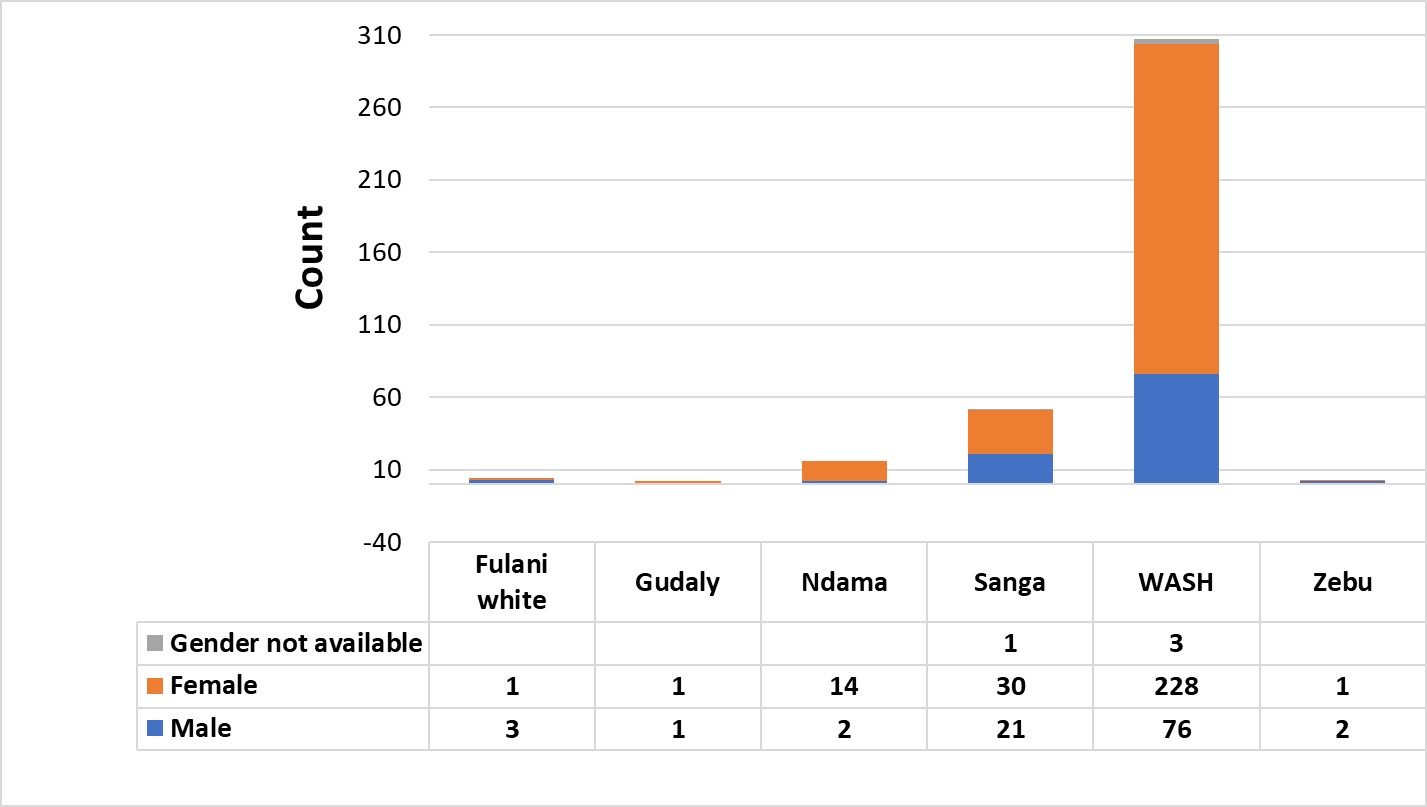


**S1 Fig**. Breeds and gender distribution of cattle sampled.

WASH: West African Short Horn

Supplement: S1 Fig — WASH: West African Short Horn. (DOCX) [file pntd.0010649.s001.docx]

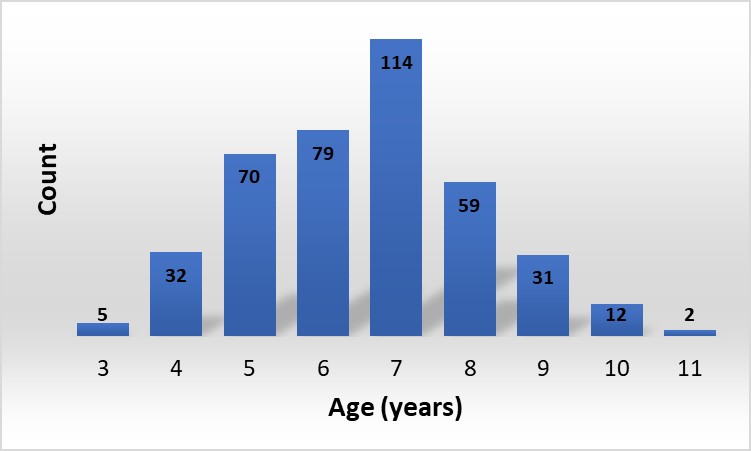


**S2 Fig**. Age distribution of cattle sampled

Supplement: S2 Fig — (DOCX) [file pntd.0010649.s002.docx]
